# Canadian Medical Association

**Published:** 1872-10-01

**Authors:** 


### Society Reports.

### CANADIAN MEDICAL ASSOCIATION.

The fifth annual meeting of this association opened in Montreal, on Wednesday, the 11th of September, in the building of the Natural History Society. The attendance of members from the Province of Quebec was fair, but the other Provinces of the Dominion were sparingly represented.

A letter was read from the President of the association, (Dr. Sewell, of Quebec,) stating that up to the last moment he was in hopes of getting away, but that the critical condition of one of his patients made it impossible for him to leave.

Dr. C. C. Hamilton, Vice-President for Nova Scotia, was called upon to preside, and Dr. McNab, from New Hampshire, was requested to take a seat upon the platform.

The first order of business was the President's address, which was read by Dr. Marsden, of Quebec, an able and interesting essay which was ordered to be printed in the transactions of the society.

A considerable portion of the time of the association was taken up in the discussion of the proposed Medical Bill, the object of which was to render the system of medical education, medical examinations, and registrations uniform throughout all the Provinces of the Dominion. The editors of the *Canada Medical Record*, in commenting on the proceedings of the society, give the following resume of the discussion :

When the time for the consideration of the Bill came round, an attempt was made to at once abandon the Bill, and thus prevent the time of the association being occupied by its discussion. At Ottawa a large representation from the Province of Ontario fully expressed themselves upon the Bill ; for instance striking out the Branch Councils and substituting one large Central Examining Board. At Quebec, owing to the amendments which had been made at Ottawa not having been translated into French and embodied in the Bill, its discussion was postponed till the present meeting in Montreal, so that, to say the very least, the association was bound, in all fairness to those gentlemen who had labored so arduously upon the Bill Committee, to give time for an expression of opinion by the members in the Province of Quebec.

The resolution to abandon the Bill was lost, and the meeting at once entered upon its discussion. The French speaking members of the association freely stated their opinion, which was that, while approving of much in the Bill, they were opposed to handing over medical legislation to the Federal Government. A resolution to that effect was proposed by Dr. Rottot, an influential representative of our French Canadian brethren, and although it was lost by a small majority, still the expression of opinion was so universal and decided, that when the association met the following morning, Dr. Howard, Chairman of the Committee who had charge of the Bill, asked leave to withdraw it. To this the association would not consent, but, by unanimous agreement, its discussion was postponed for two years, the Bill still remaining in the hands of the same Committee.

While we regret that the result is as stated, we cannot express surprise, for all must have seen that, with the French element opposed to the Bill, it was an impossibility to carry it through the Parliament of the Province of Quebec. This being the case, its abandonment, for the present at all events, was a necessity; still it would have been a great pity to have been forced to do so, without having given an opportunity to that section of our medical population who, up to the present meeting, have had no fair means of thoroughly understanding the proposed Act. The discussion was conducted with the best of taste and with the utmost good feeling, and we trust that, though the French and English members of the profession differ at the present moment, upon this subject, the time is coming when such will not be the case—when the entire profession of the Province will unite hand in hand, and press the measure to a successful termination. Our own opinion is that it only requires time to convince all of the desirability of a Dominion Act. The universality of medicine shows that those who practice it should not be confined by Provincial boundaries. The barriers which now surround each Province can only be removed by mutual concessions, and, in breaking these down, we hope the Province of Quebec will eventually take a prominent part. The object to be obtained — the right of all Canadian graduates to practice from the Atlantic to the Pacific —is one well worth sacrificing minor details for; that all will see this before long we feel convinced.

No inconsiderable portion of the meeting was, however, occupied by purely scientific papers and discussion, which gave to the present gathering an interest and a character which none of the preceding conventions have possessed. In this respect, several members of the profession in Montreal set a good example in having papers prepared, which we have reason to believe will bear good fruit next year. The last session of the association, held on the Friday afternoon, is admitted to have been exceedingly interesting, in a professional point of view, and its action in requesting addresses upon certain subjects at the next meeting from prominent members of the profession—in this respect following the plan of the British Medical Association—will cause all to look forward with much pleasure to the Sixth Annual Convention, which takes place at St. John, New Brunswick, on the first Wednesday in August. Altogether, we consider that the association has taken a new lease of life, and that the plans proposed, to give interest and *eclat* to its future meetings are such as must succeed, and we now call upon the profession to give it their cordial support.

